# Food Calls in Common Marmosets, *Callithrix jacchus*, and Evidence That One Is Functionally Referential

**DOI:** 10.3390/ani8070099

**Published:** 2018-06-21

**Authors:** Lesley J. Rogers, Leanne Stewart, Gisela Kaplan

**Affiliations:** School of Science and Technology, University of New England, Armidale, NSW 2531, Australia; leanne.stewart27@gmail.com (L.S.); gkaplan@une.edu.au (G.K.)

**Keywords:** food calls, common marmosets, food type, referential, intentional, audience effect, playback experiment, food preferences

## Abstract

**Simple Summary:**

We studied the calls that are made by common marmosets when they see food and when they consume it. They were tested with two types of fruit (banana and blueberries) and two types of insect (live mealworms and crickets). All of these foods elicited Call A, described previously as a food call. Call B was softer and was given more often to crickets. Of particular interest was our discovery of Call C, which was produced when the marmosets discovered insects but not fruit. We showed that Call C has referential meaning, by playing recordings of the call to marmosets when they were eating banana, as follows: on hearing Call C, but not Call A, they stopped eating the banana and went to look in container where they had found insects before. Call C, therefore, has specific meaning, signaling the availability of insect food. All of the calls were produced more frequently when the marmosets were tested alone than when in pairs, indicating that they advertise the availability of food to companions. Call C, at least, is not merely an automatic response reflecting the marmoset’s emotional state. A meaningful call about the discovery of insect food would be essential for survival, since marmosets often forage out of sight of each other. Knowledge of their vocal communication can improve the care of marmosets held in captivity.

**Abstract:**

We studied three calls of common marmosets, *Callithrix jacchus*, elicited in the context of food. Call A, but not B or C, had been described previously as a food call. We presented insects (live mealworms or crickets) and fruit (banana or blueberries) and used playbacks of calls. We found that Call C was produced only in response to seeing insects, and not fruit; it consistently signaled the availability of insects (includes mealworms), and more so when this food could be seen but not consumed. Playback of Call C caused the marmosets to stop feeding on a less preferred food (banana) and, instead, go to inspect a location where mealworms had been found previously, providing evidence that it has referential meaning. No such immediate response was elicited on hearing Call A or background noise. Call A differed from C in that it was produced more frequently when the marmosets were consuming the food than when they could only see it, and call A showed no specificity between insects and fruit. Call B was emitted less frequently than the A or C calls and, by the marmosets that were tested alone, most often to crickets. An audience effect occurred, in that all three calls were emitted more often when the marmosets were tested alone than when in pairs. Recognition of the functional significance of marmoset calls can lead to improved husbandry of marmosets in captivity.

## 1. Introduction

Calls that alert conspecifics to a food source have an essential role in group-living species, and many primate and avian species are known to produce food-associated calls when locating or consuming food [1,2]. One type of food call that is produced by the common marmoset (*Callithrix jacchus*) has been described previously [3], but no previous papers have reported other food calls in this species. Since the marmosets in our colony were found to emit two other calls in addition to the call that was described by Vitale et al. [3], we decided to investigate their vocal responses to food presentation in detail, and to test the possibility that the calls might be produced in reference to specific food-types. We wanted to determine whether these calls are involuntary responses to food and/or whether they are consistently produced calls that are referential.

Calls are considered to be functionally referential if they are emitted reliably in response to a specific external event and if those hearing the calls (the receivers) react in a way that is consistent with the external event, even in its absence [4]. For example, a food call would have to be emitted reliably by the signaler when food is present, and possibly only when a specific type of food is present, and the receivers hearing this call would have to respond by searching for the food or approaching the caller and the food source [5].

It is possible that the signaler is not calling with the intention of communicating to the receivers but merely responding reflexively to an external event, and that the receivers may learn to associate this call with that particular external event; that is, the receivers may extract information from the sender’s call without the sender having any intention to inform them.

This can be further investigated by determining whether the calls are produced only in the presence of group members (i.e., the ‘audience effect’) [6]. The audience effect indicates that individuals are not simply vocalising because of a change in their level of arousal, but rather that they are able to control their vocal behaviour depending on the presence or absence of other individuals [7], and this is particularly seen in the antiphonal calling of common marmosets [8,9]. In fact, the presence of others can increase or decrease call production. This behaviour has been shown in many different primate species, including red-bellied tamarins [10], vervet monkeys [11], brown capuchins [12], tufted capuchins [13], and bonobos [14]. In chimpanzees, increased rates of food calling occur in the presence of an audience but only when a large, sharable quantity of food is available; the rate of calling decreases if the quantity of food is too small to share [15]. In short, these signals represent selective vocal responses that are designed for communication, rather than simply being a reflection of the caller’s internal state, related to level of hunger or preference for a particular type of food [16].

A study of food calls (rough grunts) in chimpanzees has shown that, when a male is feeding silently, playback of pant hoot calls, simulating the arrival of another chimpanzee, elicits food calls from the feeding male. This occurs more frequently when the pant hoot calls that are played are those of a chimpanzee of much higher rank than the chimpanzee who is feeding [4]. Hence, in this species, food calls are functionally referential signals that are produced selectively and intentionally, and they are not simply broadcast indiscriminately. Note also that the structure of food calls that are produced by chimpanzees can be modified by social influences, as demonstrated by the convergence over time of the acoustic structure of food calls that are made after integration of two different groups with, originally, different food calls [17]. This indicates that such food calls of chimpanzees have enough flexibility to be modified by social learning.

Considering New World primates, Geoffroy’s marmosets (*Callithrix geoffroyi*) have been shown to increase foraging behaviour after hearing playbacks of food-associated calls [18], and white-faced capuchins approach playbacks of food calls, but not other calls [19]. The latter approach the source of food calls more often when the duration of the call is longer. Conspecifics are also seen to approach males who are calling more often than they approach calling females, and males are also observed to call more than females. Since infant cotton-top tamarins are more likely to obtain food when an adult is emitting food-associated calls [20], food calls may also function to facilitate social learning.

Relatively little is known about the food calls of marmosets. Therefore, we considered it important to examine the calls that had been noticed by one of us (LS) to be produced by the marmosets in our colony at feeding times. One call was that which was described previously by Vitale et al. [3], and there were two others that had not been described previously. Our aim was to determine whether these three calls were produced (1) on seeing or on consuming food; (2) when the marmoset was alone or in the presence of a familiar cage-mate; and (3) to test whether one or more might be referential signals.

## 2. Materials and Methods

### 2.1. Subjects and Housing

The 12 subjects (6 male and 6 female) that were used in this study were part of a colony of common marmosets (*Callithrix jacchus*) that were housed at the University of New England, NSW, Australia (holding Permit AEC05/061). The individuals that were tested, males and females, ranged in age from 24 to 162 months. All were the offspring of captive-bred marmosets.

They were housed in same-sex pairs, in three separate home rooms (4 × 3 × 3 m), each containing three or four home-cages (size 1 × 2.3 × 2 m). These home-cages were connected by runways (wire-mesh enclosed structures of 0.23 × 0.23 m cross section and several metres in length) to three indoor rooms (3 × 2.9 × 2.6 m), and each of these was, in turn, connected to an outdoor cage (1.7 × 1.7 × 2.5 m) where the marmosets could receive exposure to sun light and observe activities in the external environment [21]. Each home-cage was richly furnished with wooden perches, branches of different sizes, hanging objects, tunnels, tyre swings, at least one nest box, and a tray containing a blanket and heat pad. The indoor rooms had horizontal and vertical branches, and each outdoor cage was furnished with several branches and a suspended pipe, which the marmosets could enter. Access to the indoor rooms and outdoor cages was rotated between the home-cages every three days. The experiments were conducted in the indoor rooms.

The rooms were kept within a temperature range of 18 °C to 30 °C. Lights came on at 07:00 h and went off at 19:00 h, and sunlight entered the home rooms via skylights. Additional ultra violet light (350–390 nm) was provided for 30 min each day in the home rooms. 

Feeding occurred daily between 12:00 h and 14:00 h. The daily diet was banana cake, meatloaf, dog pellets, apples, banana, with weekly extras of boiled egg, sultanas, yoghurt, green beans, cheese, stewed apple, nutra-grain, peanuts, wholemeal bread with vitamin supplement, gum Arabic, and fresh seasonal fruits, as well as occasionally including blue berries. Water was available ad libitum. Mealworms and crickets were given approximately once a month. Prior to conducting these experiments, and during them, the experimenter (LS) fed the marmosets twice per week to maintain familiarity and thus help to avoid stressful situations during the tests. 

Individual marmosets were identified by their cage group, as well as by characteristic features, such as size, facial markings, and other individual features. 

### 2.2. General Procedure for Testing

The University of New England approved the experimental procedures (AEC08/034). All of the experiments were conducted in the indoor rooms between 09:00 and 12:00 h, before the daily feeding time. The indoor rooms were accessed by the marmosets via the runways, which had a number of sliding panels that could be closed to prevent retreat to the home cage or access to the outdoor cage. One-way mirrors from an observation room permitted the experimenter to remain unseen while recording the marmosets’ behaviour during testing.

Four types of food were used in testing, namely, two fruits (banana and blueberries) and two insects (live mealworms and crickets). The food was presented on a wooden table (60 × 90 × 75 cm), covered with hessian, and placed in the middle of the indoor room.

The recording of the vocalizations was made using a Sennheiser microphone and a Marantz digital recorder. Video footage was obtained using a JVC mini DV recorder. All of the equipment was operated remotely. The recording microphone was placed in the indoor rooms 24 h before testing so as to ensure that the marmosets were familiar with all of the equipment that was used. The behaviour of the marmosets was also videotaped. The video camera was situated in the left corner of the indoor room on a tripod.

#### 2.2.1. Call Analysis

The calls were identified from sonograms using Raven, a bioacoustics program that was developed by the Cornell Bioacoustics Research Program, and Adobe Audition 2.0. The frequency, duration, pitch, and amplitude were assessed.

#### 2.2.2. Statistical Analysis

Normally distributed data were analysed by one-way ANOVA using SPSS Statistics program 17.0. The data that could not be normalised by transformation were analysed using non-parametric tests.

### 2.3. Experiment 1: Determining the Relative Preferences of Food Types Used in Subsequent Tests

Before assessing the vocal responses of the marmosets to food, tests were made to determine the relative preferences of the marmosets for the four food types that were used in subsequent tests. A set of paired choice tests was given prior to conducting Experiments 2 and 3, and a second set of choice tests was given after the completion of Experiments 2 and 3.

Twelve marmosets (6 males and 6 females) were tested in the indoor rooms. Petri dishes (75 mm diameter, height 5 mm) were used in this experiment for easy visibility of the food types, because it was necessary for the marmoset that was being tested to see both of the food types that were presented at the same time, in order to make a choice. These dishes were attached to the sheet of Perspex using small pieces of Velcro, so as to stop the movement of the dishes and to allow easy removal. The marmoset could take only one piece of food per trial and the dishes were removed once a choice had been made. In each choice test two semi-randomly assigned food types were presented, and in small quantities (one mealworm, one cricket, one blueberry, and a 1 cm cube of banana). During each presentation, the individuals were considered to have made a choice when they had observed both dishes, by looking directly at each food type, and had then approached and taken food from one dish. Once a choice had been made, the experimenter removed both of the dishes and left the room to change them, before returning for the next presentation. 

Each trial consisted of paired combinations of food types. One set of choices was run per day and a score of 1 was recorded for the food that was chosen in each choice test. 

### 2.4. Experiment 2: Vocal Responses of Marmosets Tested in Pairs

This experiment was designed to observe the vocal response of familial pairs of marmosets to four different food types. The calls were recorded during three conditions, namely, (1) no food present; (2) food presented in a bowl with a transparent lid so that it could be observed but not eaten; and (3) the lid removed from the bowl and the food available to be eaten. The aim was to observe whether particular calls were given in the presence of food, and if different calls were elicited by different food types.

The marmosets were tested using home-cage mates (*N* = 12 marmosets, the same as in Experiment 1; 6 same-sex pairs of which 3 were female and 3 were male). The pairs received one presentation of each type of food per day, in random order and in large quantities; half a banana (chopped), 20 blueberries, 20 mealworms, and 10 crickets (i.e., approximately the same amount of each food type). All of the food was presented in large amounts, as previous research had shown that higher rates of food calling were elicited by larger amounts of food (marmosets [3]; red-bellied tamarins [10]). 

The food was presented in white ceramic bowls (diameter 9.5 cm, height 7 cm) that were covered with a transparent plastic lid (diameter 9 cm). Each food type had its own bowl to avoid any olfactory cues being transferred across the different types of food. The bowl containing food was placed on the table in the indoor room. 

The recording sessions consisted of a six-minute period prior to testing (no food present in the indoor room), followed by a three-minute period when the food was presented in a bowl with a transparent lid, to allow the marmosets to observe, but not eat it. This was followed by a three-minute period after the lid had been removed from the bowl, so that marmosets could access the food. 

#### Identification of Calls

Three calls (A, B, and C), which were previously noted to be produced in the presence of food, could be distinguished accurately from the phee and tsik calls in structure and frequency, and from the other common and known calls of marmosets [22]. Since it was not possible to distinguish which individual made each call, the total scores were determined for each pair of marmosets.

**Figure 1:**
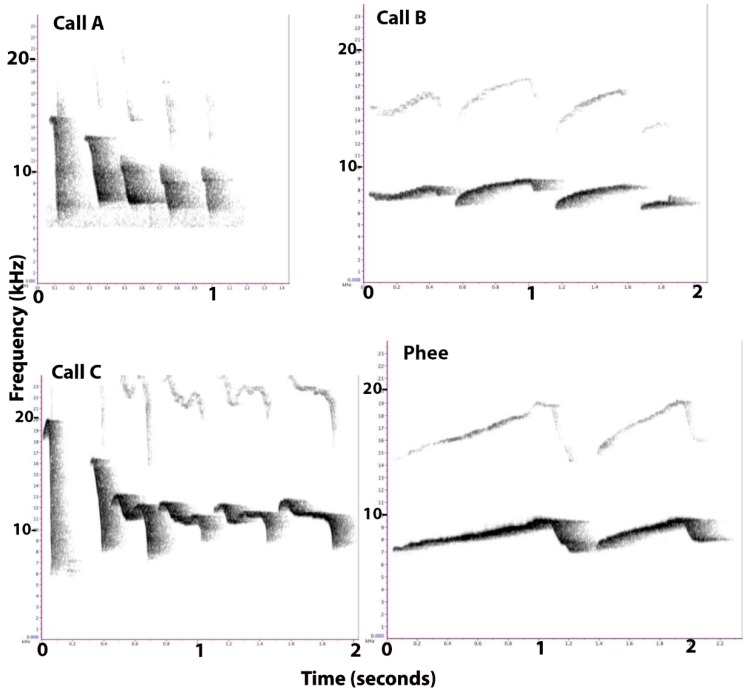
Representative sonograms of Calls A, B, and C, and, for comparison, of a phee call. Frequency in kilohertz (kHz) is plotted on the *y*-axes and the duration, in seconds, is plotted on the *x*-axes. Note that the scale differs slightly for each call type.

As a result of their entirely different structures (frequency, duration, and rhythm), Calls A, B, and C were easily distinguishable in sonograms (as well as by ear). All three of the calls were relatively stereotyped and could be readily identified (Figure 1). 

Call A consisted of short, loud, chirping sounds that were uttered consecutively with 4–7 chirps/second, with a duration ranging from a few chirps to a sequence lasting 6–8 s. The frequency of this call ranged from 4–11 kHz, usually beginning with one ‘tsik’ call or sometimes two (Figure 1, Calls A). Each repeated element of the call had a descending frequency and was of a duration of approximately 0.08 to 0.10 s. This call was described previously as a food call by Vitale et al. [3].

Call B was a succession of 4–6 soft whistles, each of a 0.5 s duration, given with the mouth almost closed. It had a duration of approximately 2 s (four whistles), and a frequency ranging from 6–9 kHz (Figure 1, Calls B). The amplitude of the B calls was quite low and the frequency contours were relatively flat. Although the structure of this call looked like a phee call (Figure 1, Phee), the average frequency of a phee call is 8–10 kHz, with call duration averaging 1.5 s, and it is a loud, long distance contact call [22]. The frequency of Call B was lower than that of a phee call, and the amplitude was much lower. The individual elements of phee calls also have a longer duration (1–2 s) than those of the B call (0.5 s). Call B was given in groups of 4–5 elements, whereas the phee calls usually contain 2–3 elements. In most cases, Call B had a trill in all or some of the syllables, appearing similar to a trill-phee, as described by Pistorio et al. [23]. 

Call C was a sequence of long, loud squeals, beginning with a single ‘tsik’ vocalization, peaking at 20 kHz, and often followed by a second quite similar element, peaking at 16–17 kHz. Subsequent elements ranged from approximately 8 to 13 kHz and dipped midway to about 11 kHz, so that they had an approximate m-shape (Figure 1, Calls C). Each element had a duration of about 0.35 s. The call had a duration of 6–8 s, and the frequency ranged from 6–16 kHz, including the second element, but not the first, tsik element. 

### 2.5. Experiment 3: Vocal Responses of Marmosets Tested in Isolation

This experiment measured the vocal responses of individual marmosets to the four different food types when they were tested alone in the indoor rooms. The number of each type of call could be determined and compared to the number of calls that were made in pairs.

The testing procedure and apparatus that were used were the same as that which was used in Experiment 2, except that the twelve marmosets were now tested singly. Although they could hear the louder calls that were made by other members of the colony in the home rooms, these were muted. No visual contact with other marmosets was possible.

### 2.6. Experiment 4: Response to Playback of Calls A and C

Experiment 4 was designed to test whether the A or C calls had a referential quality signalling the presence of ‘insects’ or ‘live food’. The aim was to playback the calls to see whether they elicited a specific response in the absence of the particular food to which they might have referred. Based on the results of Experiments 2 and 3, Call B was not tested in this way.

The marmosets were trained, prior to conducting the experiment, to find mealworms in a cup that was attached behind a branch in the far corner of the indoor room (1.5–1.8 m from the table). Mealworms were placed in the cup each afternoon, and when no marmoset was in the room, for at least four days prior to the commencement of the test trials. The cups were baited once per day after the marmosets had returned to their home rooms at feeding time and, since the marmosets had access to the indoor rooms via the runways, they could find the mealworms when they next entered this room. Since the mealworms were not visible from a distance, the marmosets had to inspect the cup to discover the mealworms.

Once the marmosets had taken the mealworms from the cup on three consecutive days, testing began. At testing, the marmosets were assigned randomly to receive one of the three playback sequences, namely, (1) Call A; (2) Call C; and (3) background noise from the animal house. No mealworms were in the cup before or during the experiment. The marmoset that was being tested was given a bowl of banana on the table in the indoor room. The banana was presented in the bowl on the table and was mashed to ensure that the marmoset that was being tested remained at the bowl at least initially during the playbacks, and could not pick up a piece of banana and move away from the table. The playback sequences of the calls were presented once the marmoset had begun to eat the banana and had done so for a minimum of 30 s. If a call indicated that a more preferred food was available, or even more specifically, that insects were available, it was predicted that, on hearing this call, the marmoset should leave the bowl containing the banana and go to look for the mealworms in the cup at the far corner of the room.

Either Call A or Call C was played back via a speaker that was placed 1 m from the bowl of banana, and approximately 4 m from the bowl of mealworms. Call A was chosen to be compared to Call C, because the previous experiments had shown that Call A was a non-specific call that was given to all of the food types, and mainly during consumption of food. It was also necessary to playback a control sound, which was a background noise that was recorded in the animal house.

#### 2.6.1. Playback Sequences

All of the playback sequences were taken from recordings that were made during Experiment 3 using a Sennheiser microphone and a Marantz audio recorder. The Call A sequences were derived from recordings that were made when fruit had been presented, and the Call C sequences were taken from the recordings when either mealworms or crickets had been presented. Individuals were played sequences that had been recorded from their cage mate only. Playback of background noise from the animal house consisted largely of the sounds of an air conditioner and other noises that were familiar to the marmosets. No calls of any kind were present in this recording and it was 55 s in duration.

The playback sequences of Call A were arranged using Adobe Audition, by extracting nine individual calls of type A and arranging each of them in a 55 s sequence, with 1.0, 3.0, or 10 s of silence between calls, and in that sequence of occurrence. The same procedure was used to prepare the playback sequences of Call C. These sequences were then cleaned of background noise and other sounds using Raven. All of the playback sequences were calibrated to 65 decibels at a distance of 1.0 m from the speaker. 

#### 2.6.2. Subjects and Recording of Behavior

Each marmoset was tested in isolation. The same marmosets as those that were used in the previous experiments were tested, excepting one male. The call sequences were played back through a speaker that was placed next to the table (1 m from the bowl of banana), facing towards the back of the room. The playbacks were presented via a Behringer 2-way active ribbon studio reference monitor with a Kevlar woofer Model B3030A, from an Apple iPod shuffle. Only one playback was presented per day and the order of the calls and the control sound was pseudo-randomised so that the playback sequence of any call type was not given twice in a row. The tests were repeated once for each marmoset (Test 1 and Test 2, separated by an interval of one month). The behaviour of the marmoset was video recorded.

#### 2.6.3. Scoring

The behaviour was scored during 4 min of testing, starting from the beginning of the playback. The latency from the time of commencing playback to the first inspection of the cup was measured. As a strict criterion was used for scoring the inspection, the marmoset had to look directly into the cup and not just approach the cup. Marmosets often put their faces inside the cup when inspecting. The marmosets that did not inspect the cup during the test period were scored as a maximum latency of 240 s.

## 3. Results

### 3.1. Experiment 1: Determining the Relative Preferences of Food Types Used in Subsequent Tests

**Figure 2:**
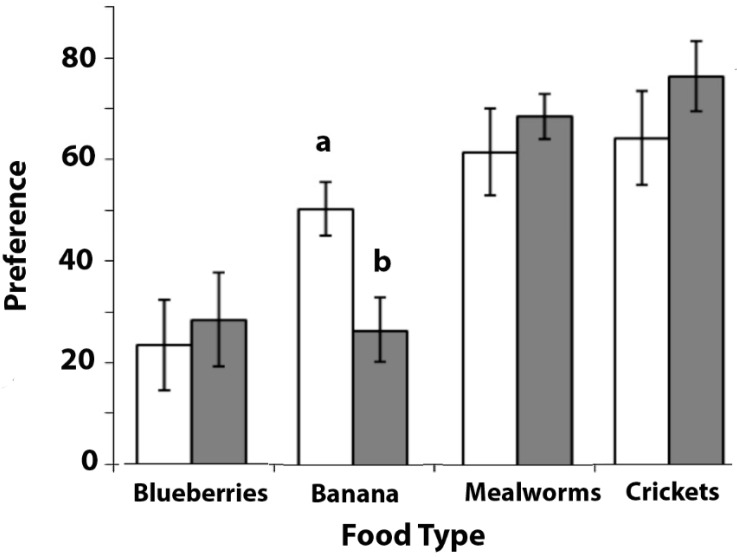
The results of Experiment 1. Choice of each food type is presented as a mean with standard error bars. *N* = 12. White bars are for the first preference test and dark grey bars or the second preference test (see text for details). The bar marked with (**a**) is significantly different from that marked with (**b**).

The choice scores (i.e., preference) for the four food types were calculated (Figure 2).

The scores for the first set of choice trials, which were conducted before the main experiments, were arcsine transformed using asin(√n)x 52.298, as recommended by Zar [24], and were then analysed by one-way ANOVA with the food type as a repeated measure. A significant difference between the preferences for the different food types was found (F_(3, 24)_ = 4.231, *p* = 0.016). Post hoc comparisons revealed that there was a tendency towards a higher preference for banana over blueberries (Least Significant Difference (LSD): *p* = 0.051), a significantly higher preference for mealworms over blueberries (LSD: *p* = 0.024), and for crickets over blueberries (LSD: *p* = 0.034). The blueberries were preferred the least of all of the food types. There were no significant differences between the preferences for banana versus mealworms (LSD: *p* = 0.272), banana versus crickets (LSD: *p* = 0.242), or between mealworms versus crickets (LSD: *p* = 0.909). 

Since the scores for the second set of trials, which were conducted after completing Experiments 2, 3, and 4, could not be normalised by transformation, non-parametric tests were used. A significant heterogeneity was found in the preferences for the different food types (Friedman test: χ^2^ = 11.830, *N* = 9, *p* = 0.008). Post hoc comparisons (Wilcoxon Signed Ranks Tests) revealed that there were significantly higher preferences for mealworms over blueberries (Z= −2.310, *p* = 0.021) and mealworms over banana (Z = −2.666, *p* = 0.008), as well as for crickets over blueberries (Z = −2.240, *p* = 0.025) and crickets over banana (Z = −2.240, *p* = 0.025). There were no significant differences between the preferences for banana and blueberries (Z = 0.0, *p* = 1.0), or between mealworms and crickets (Z = 0.059, *p* = 0.953).

A comparison between the first and second data sets showed that banana was preferred more in the first testing period compared with those in the second testing period (Wilcoxon Signed Ranks, Z = −2.521, *p* = 0.012). No significant change occurred in the preferences for blueberries (Z = −0.674, *p* = 0.500), mealworms (Z = −0.338, *p* = 0.735), or crickets (Z = −0.350, *p* = 0.726).

The marmosets chose insects, either crickets or mealworms, by preference over fruit, banana or blue berries. Except for banana, the preferences for the food types were consistent across the two tests.

### 3.2. Experiment 2: Vocal Responses of Marmosets Tested in Pairs

**Figure 3:**
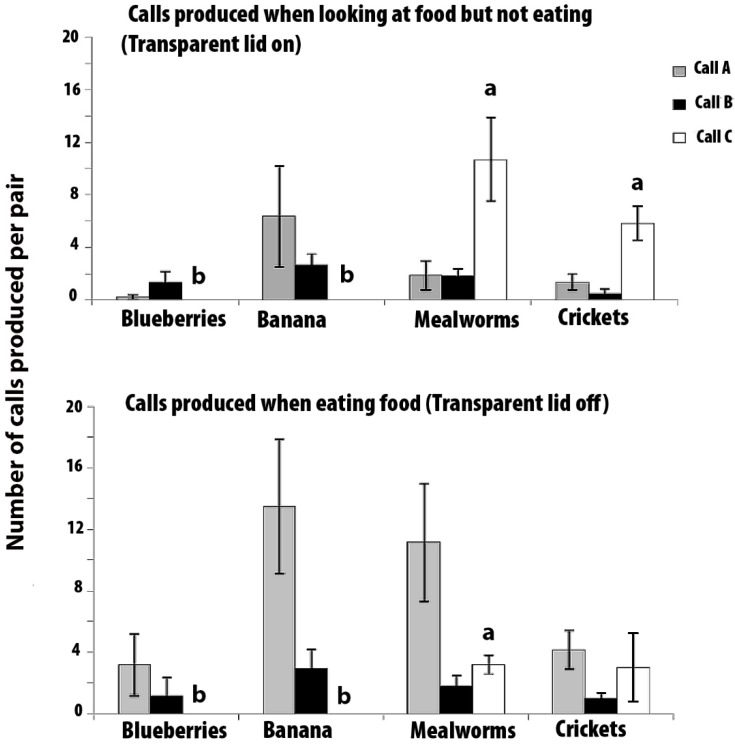
Experiment 2. The mean number of calls produced during the 3 min periods with the transparent lid-on (top graph) and lid-off (bottom graph). The data are for marmosets tested in pairs (*N* = 6 pairs). Standard error bars are indicated. Grey bars for Call A, black bars for Call B, and white bars for Call C. Bars marked with (**a**) differ significantly from those marked with (**b**).

The mean numbers of calls that were produced (±SEMs) per pair in 3 min with the lid on and 3 min with the lid off are presented in Figure 3. No calls of type A, B, or C were produced during the 6 min pre-test period, although some phee calls were heard during this period. Some trills were heard when the experimenter entered the room with the food bowl, but Calls A, B, and C were produced only when the food was presented. Note that Calls A and B were made in the presence of all of the food types, but call C was produced only when the mealworms and crickets were presented.

Non-parametric tests were used as the data could not be normalised by transformation. The data were first analysed for heterogeneity using Friedman’s Tests for each call type, and separately in the lid-on and lid-off conditions, with the food type as the factor. If significant heterogeneity was found, post hoc tests (Wilcoxon Signed Ranks tests) were applied to locate the differences between the food types. Comparisons between the lid-on and the lid-off conditions were made using Wilcoxon Signed Ranks tests. *N* = 6, since 6 pairs were tested.

Call A: There was no significant heterogeneity with respect to the food type in the number of A calls in the lid-on condition (Friedman Test, χ^2^ = 4.143, *p* = 0.246: Figure 3 top graph), or in the lid-off condition (χ^2^ = 4.636, *p* = 0.200: Figure 3 bottom graph). Hence, these scores were lumped across the food type to compare the lid-on versus lid-off scores. There were significantly more A calls made in the lid-off condition than the lid-on condition (Z = −3.291, *p* = 0.001). In other words, Call A was produced more often when the marmosets had access to the food than when they could see but not eat it. 

Call B: There was no significant effect of food type in the number of B calls in the lid-on condition (χ^2^ = 7.286, *p* = 0.063) or in the lid-off condition (χ^2^ = 4.059, *p* = 0.255). Hence, the number of B calls were lumped across the food type to compare the lid-on versus lid-off condition, but no significant difference was found between these two conditions (Z = −0.242, *p* = 0.808). Hence, although no B calls were produced unless food was present, the call was non-specific for food type or whether or not the food was accessible. 

Call C: During the lid-on condition, there was a significant difference between the number of C calls that were elicited by the presentation of the different foods (χ^2^ = 16.22, *p* = 0.001), and also in the lid-off condition (χ^2^ = 13.50, *p* = 0.004). No C calls were elicited by either blueberries or banana. Post hoc tests using Wilcoxon Signed Ranks tests revealed that, in the lid-on condition, significantly more C calls were produced when mealworms could be seen than when blueberries (Z = −2.207, *p* = 0.027) or banana could be seen (Z = −2.207, *p* = 0.027). Also, significantly more C calls were elicited by crickets than by blueberries (Z = −2.226, *p* = 0.026) or banana (Z = −2.226, *p* = 0.026). There was no significant difference between the number of C calls that were given for crickets and mealworms (Z = −1.153, *p* = 0.249). 

In the lid-off condition, post hoc tests revealed that significantly more C calls were produced when mealworms were presented than when blueberries (Z = −2.232, *p* = 0.026) or banana (Z = −2.232, *p* = 0.026) were presented (i.e., the scores in the mealworm presentations were significantly above zero, and no C calls were produced for the presentations of blueberries or banana). C calls were produced when access to crickets was possible, but the response was variable and none of the comparisons to C calls that were elicited by the other foods were significant (crickets versus mealworms, Z = −0.677, *p* = 0.498; crickets versus banana, Z = −1.633, *p* = 0.102; and crickets versus blueberries, Z = −1.633, *p* = 0.102).

There were significantly more C calls given to mealworms in the lid-on condition than in the lid-off condition (Z = −1.992, *p* = 0.046). There was no significant difference between the number of C calls that were given in response to seeing crickets in either the lid-on or the lid-off conditions (Z = −1.687, *p* = 0.092). 

Hence, for mealworms, Call C was produced more frequently when the lid was on than when the lid was off, and it is note that this was opposite to the result for Call A.

### 3.3. Experiment 3: Vocal Responses of Marmosets Tested in Isolation

**Figure 4:**
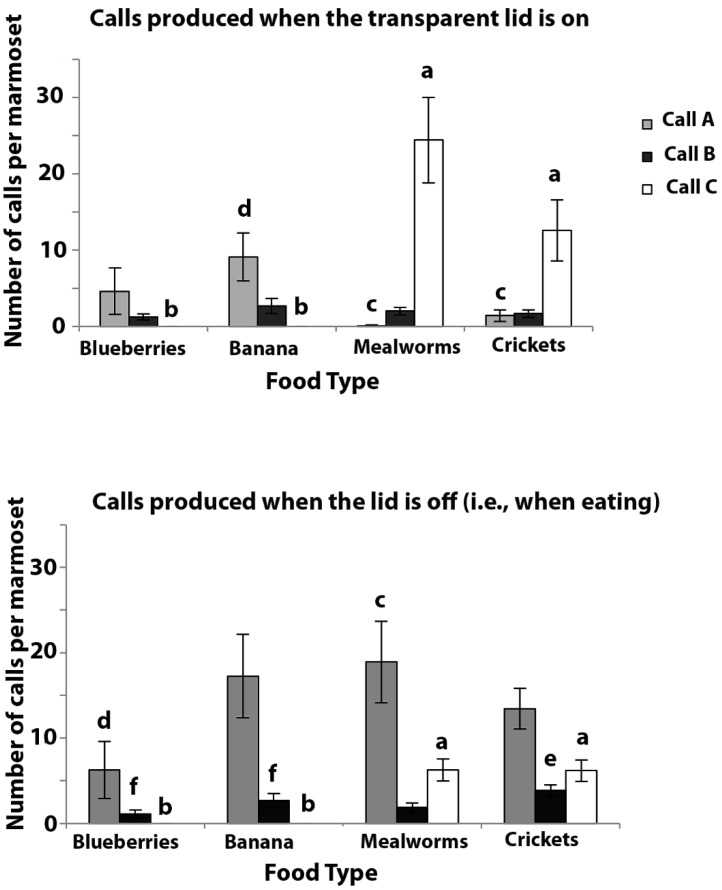
Experiment 3. These data are presented as in Figure 3 but here each marmoset was tested alone and *N* = 12. Bars marked with (**a**) differ significantly from those marked with (**b**), those marked (**c**) differ significantly from those marked (**d**) and similarly for those marked (**e**) versus (**f**).

The results of Experiment 3 are presented in Figure 4. This figure does not include the control period prior to presentation of the food, as no A, B, or C calls were made during this period.

Call A: Non-parametric tests were used, as the data could not be normalised by transformation. There were significant differences between the number of A calls that were given for different foods during the lid-on condition (Friedman Test: χ^2^ = 10.481, *p* = 0.015) and also the lid-off condition (χ^2^ = 7.907, *p* = 0.048). The post hoc Wilcoxon Signed Ranks test revealed that, during the lid-on condition, there were significantly more A calls that were produced when the marmosets could see banana than when they could see mealworms (Z = −2.521, *p* = 0.012) or crickets (Z = −2.310, *p* = 0.021). However, no significant difference was found between the banana and blueberries (Z = −1.245, *p* = 0.213), blueberries and mealworms (Z = −1.761, *p* = 0.078), blueberries and crickets (Z = −0.211, *p* = 0.833), and mealworms and crickets (Z = −1.841, *p* = 0.066).

During the lid-off condition, significantly more A calls were made when the mealworms could be accessed compared to the blueberries (Z = −2.355, *p* = 0.019), but none of the other comparisons were significant (crickets versus blueberries, Z = −1.690, *p* = 0.091; banana versus blueberries, Z = −1.468, *p* = 0.142; banana versus mealworms, Z = −0.865, *p* = 0.387; banana versus crickets, Z = −0.707, *p* = 0.480; and mealworms versus crickets, Z = −1.255, *p* = 0.209). 

There were significantly more A calls given in the lid-off condition compared with the lid-on condition for banana (Z = −2.805, *p* = 0.005), mealworms (Z = −3.062, *p* = 0.002), and crickets (Z = −2.936, *p* = 0.003), but this was not the case for blueberries (Z = −1.461, *p* = 0.144), which elicited fewer A calls than any of the other food types.

Call B: Fewer B calls were given than either of the A calls or C calls. The scores of the B calls were analysed by ANOVA as they could be successfully normalised by log transformation. The factors that were used in the ANOVA were the food type as a repeated measure, and lid-on versus lid-off as a factor. There was a significant difference between the number of B calls that were given for different foods (F_(3,33)_ = 3.546, *p* = 0.025), but no significant main effect for the lid-on versus lid-off conditions (F_(1,11)_ = 0.011, *p* = 0.919). However, there was a significant interaction between the two factors (F_(3,33)_ = 5.988, *p* = 0.002). The post hoc LSD comparisons revealed that significantly more B calls were produced when the marmosets had access to crickets compared with blueberries (*p* = 0.029), and to crickets compared with banana (*p* = 0.035). No significant differences were found in the tests with crickets versus mealworms (*p* = 0.209), banana versus mealworms (*p* = 0.356), blueberries versus mealworms (*p* = 0.114), or blueberries versus banana (*p* = 0.294). 

Hence, more B calls were produced when the marmosets had access to crickets compared with the other three food-types.

Call C: The data were analysed by ANOVA as they could be successfully normalised by log transformation. The factors that were used in the ANOVA were the food type as a repeated measure, and the lid-on versus lid-off conditions as the factor. There was a significant main effect of the lid-on versus lid-off condition (F_(1,11)_ = 12.136, *p* = 0.005), and a significant interaction between the two factors (F_(3,33)_ = 6.130, *p* = 0.002). There was also a significant difference between the number of C calls that were given for the different foods (F_(3,33)_ = 56.773, *p* < 0.0005). Pairwise LSD comparisons revealed that significantly more C calls were produced on seeing mealworms compared with blueberries (*p* < 0.0005), mealworms compared with banana (*p* < 0.0005), crickets compared with blueberries (*p* < 0.0005), and crickets compared with banana (*p* < 0.0005). However, there was no significant difference between the number of C calls that were produced on the presentation of mealworms or crickets (*p* = 0.419). No C calls were elicited by banana or blueberries. 

Post-hoc tests showed that there were significantly more C calls given to mealworms during the lid-on than the lid-off condition (paired *t*-test: *t* = 4.369, *p* = 0.001), although this was not the case for crickets (paired *t*-test: *t* = 0.783, *p* = 0.450). 

In summary, C calls were elicited only by mealworms and crickets, as found in Experiment 1, and more C calls were produced when the marmosets could simply see the mealworms than when they had access to them; note that this is the opposite to the results that were found for Calls A and B.

#### 3.3.1. Correlation of Preference for a Food and the Number of Calls Elicited

The relationship between the number of calls that were emitted by individuals to the four food types in Experiment 3 was correlated with the percent of the food preference for each food type that was determined in Experiment 1, using Pearson’s correlation test. The preferences that were determined in the two periods of testing in Experiment 1 were combined. These data were continuous (i.e., not polarised).

A significant and strong negative correlation was found between percent preference for mealworms and the number of A calls that were given during the lid-off condition (*r* = −0.818, *p* = 0.007). For Call B, the percent preference for blueberries correlated positively with the number of calls that were produced upon seeing blueberries with the lid on (*r* = 0.768, *p* = 0.016). For Call C, the number of calls that were produced correlated negatively with the preference for mealworms in the lid-on condition (*r* = −0.729, *p* = 0.026) and the lid-off condition (*r* = −0.794, *p* = 0.011). No other correlations were significant (*r* values ranged from −0.571 to 0.553, *p*-values ranged from 0.108 to 0.806).

#### 3.3.2. The Number Calls Given to Food by Marmosets when in Pairs and when Alone (i.e., Experiment 2 Compared to Experiment 3)

**Figure 5:**
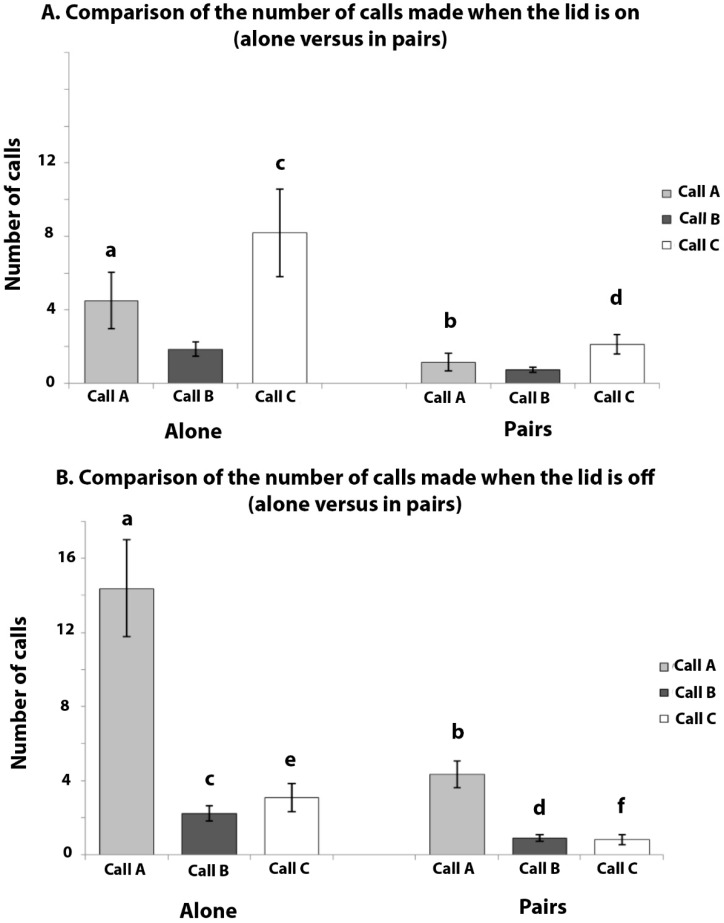
Comparison of the number of calls made in each 3 min period when tested alone compared to in pairs. (**A**) Lid-on condition. (**B**) Lid-off condition. Means are plotted and standard errors are indicated. Bars with (**a**) differ significantly from those with (**b**), (**c**) from (**d**), and (**e**) from (**f**). Although no C calls were elicited by banana or blueberries, the mean scores that are plotted included all of the presentations (insects and fruit). However, an analysis of the data did not include the zero scores for the banana and blueberries: see text.

Comparisons were made to see how the calling rate was affected by the presence or absence of a social companion. In order to do this, the scores for the number of calls were collapsed across the food types. The lid-on and lid-off conditions were considered separately. In each comparison, two tailed paired *t*-tests were applied. As the individuals could not be identified using audio recordings of their calls for the paired tests (Experiment 2), the scores of the pairs were divided by two and were compared to scores of the individuals that were tested alone (Figure 5).

Call A: In the lid-on condition, significantly more A calls were made per marmoset when tested alone, compared to in pairs (*p* = 0.035), and the same was the case for the lid-off condition (*p* = 0.016). 

Call B: During the lid-on condition there was no significant difference between the number of B calls that were made when the marmosets were tested in pairs, compared to when they were tested alone (*p* = 0.059), although the trend to make more calls when isolated was noted. In the lid-off condition, the difference was significant (*p* = 0.026). The marmosets produced more B calls when alone and eating food than when in pairs and eating food.

Call C: Since no C calls were recorded when the blueberries or banana were presented, for statistical analysis, the scores for this call were collapsed only across the tests in which the mealworms and crickets were presented. When these insects were presented with the lid on, significantly more C calls were emitted when the marmosets were tested alone, compared with when they were tested in pairs (*p* = 0.002). In the lid-off condition, fewer C calls were emitted but, despite this, significantly more C calls were given when the marmosets were tested alone compared with when they were tested in pairs (*p* = 0.0007).

Hence, significantly more A, B, and C calls were emitted when the marmosets were tested alone than when they were tested in pairs. 

### 3.4. Experiment 4: Response to Playback of Calls A and C

**Figure 6:**
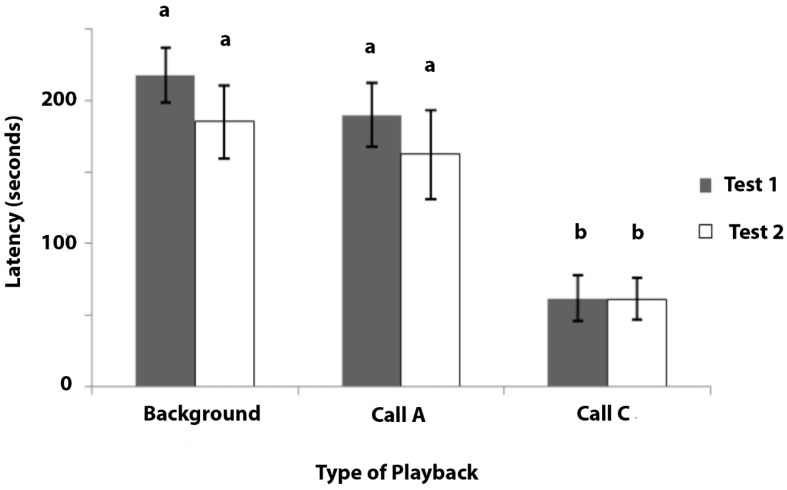
Experiment 4. Mean latency in seconds from the commencement of the playback to the first inspection of the cup (for mealworms) is plotted with standard errors indicated. The playbacks were background noise that was recorded in the animal house, Call A, and Call C. Bars marked (**a**) are significantly different from those marked (**b**). *N* = 7. Maximum response latency was 240 s.

The data of latency to inspect the cup during the three types of playback is presented in Figure 6. As these data could not be normalised by transformation, non-parametric tests were conducted. Of the 11 marmosets that were tested, 7 inspected the cup during testing, whereas 4 of the marmosets gave no response to any playback sequence during test 1 or test 2. Since these four individuals were non-responsive in this experiment, they were not included in the analysis.

Test 1: Significant heterogeneity was found in the latency to inspect the cup when hearing the different playbacks during test 1 (Friedman’s test: χ^2^ = 13.040, *N* = 7, *p* = 0.001). Post hoc tests revealed that there were no significant differences in the latency between the playbacks of background noise and Call A (Wilcoxon Signed Ranks tests: Z = −1.826, *p* = 0.068: Figure 6). There was a significant difference between the latencies during the playbacks of background noise and Call C (Z = −2.366, *p* = 0.018), and also between the playbacks of Call A and Call C (Z = −2.366, *p* = 0.018).

Test 2: Significant heterogeneity was found in the latency to inspect the cup, depending on the playback that was presented (Friedman’s test: χ^2^ = 8.857, *p* = 0.012). Post hoc tests showed no significant difference in the latency to inspect the cup between tests with background noise and Call A (Wilcoxon Signed Ranks tests: Z = −0.676, *p* = 0.499), but there was a significant difference between the background noise and Call C (Z = −2.371, *p* = 0.018), and also between the Call A and Call C playbacks (Z = −2.197, *p* = 0.028: Figure 6).

In summary, the marmosets checked the cup after a short latency on hearing playbacks of Call C, whereas they rarely inspected the cup on hearing either the background noise or Call A and, on those occasions when they did so, it was after a much longer latency than when they had heard Call C.

## 4. Discussion

The three calls (A, B, and C) were made only in the presence of food and not in the 6 min prior to the presentation of the food. Only one of these calls, Call A, had been reported previously to be a food call of the common marmoset [3]. Our results indicate that marmosets have more than one food call, as is the case in other primates and closely related species, such as Geoffroy’s marmosets [18], cotton-top tamarins [25,26], red-bellied tamarins [10], and golden lion tamarins [27], but had not been reported previously in common marmosets.

Call A was emitted in the presence of all of the food types that were tested, fruit and insects, and more frequently when the food was being consumed, confirming the previous report by Vitale et al. [3]. Previous studies showed that food-associated calls were often positively correlated with a preference for a particular food [26,28], but there was no consistent association between the number of A calls that were produced and the measured order of the food preferences in our experiments, except for significantly more A calls that were produced when eating mealworms than when eating blueberries, and only when the marmosets were tested alone. In the lid-on condition, the marmosets that were tested alone produced more A calls when looking at banana compared to looking at either mealworms or crickets. Despite these differences, as seen in Figure 4, the number of A calls that were produced did not discriminate between fruit versus insects. This led us to conclude that Call A was nonspecific for the food type. Instead, the frequency of producing A calls may have been influenced by the arousal and recognition of the food. The function of Call A may have been to solicit group members to a food source, since the marmosets made these calls more frequently when they were tested alone compared with when they were tested in pairs, as found previously by Vitale et al. [3]. However, as the correlations showed, the more the marmosets preferred the mealworms, the fewer A calls they made when they had access to them (lid-off condition). It seemed that the consumption of the preferred food suppressed the production of Call A (discussed more below).

From the results that were obtained, it was clear that Call B was produced less often than Calls A and C. The only significant findings about Call B were that (1) it was produced more often when the crickets were available in the lid-off condition; and, (2) although blueberries were the least favoured food type that was presented, significantly more B calls were vocalised the stronger the marmoset’s preference for blueberries was, but only in the lid-on condition. These significant results indicated that Call B was a food call but it was difficult to draw any other firm conclusions. The intensity of the B calls was more similar to that of a close contact call (i.e., the trill [22]) and B calls were emitted at much lower intensities than the A or C calls. Hence, Call B may have been a close contact call that was given during feeding rather than a call that solicited others to a food source. Since no significant positive (or negative) correlations were found between the preferences for the other food types and the number of B calls that were emitted, it would be premature to draw any firm conclusion about this call. Further research will be required to decide on the function of Call B, although its low intensity suggests that it would have limited, if any, capacity to act as a referential signal, especially since marmosets tend to forage in sound-attenuating natural conditions.

One of the clear findings of our study was that C calls were produced only when the marmosets were presented with mealworms and crickets, and that these calls were not emitted when fruit, banana or blueberries, was presented. The C calls were also given more often when the marmosets were observing mealworms and crickets, than when they were eating them. This result was opposite to that which was obtained for Calls A and B, suggesting that C calls may be given in response to discovering insects, or in anticipation of eating them. Marmosets in the natural environment consume insects as a large part of their diet, as these are an important source of protein [29]. Call C may, therefore, attract conspecifics to the discovery of an important dietary source.

To test whether Call C could be a referential signal, the responses of the marmosets were scored during the playbacks of the call. If the marmosets responded to hearing the playbacks of the C calls by searching for insects, this would show that this call signals information about the availability of insects and, therefore, functions as a referential signal. As shown in Experiment 4, marmosets responded to hearing the playbacks of C calls by looking for mealworms in a location and a container that was known to contain mealworms on previous occasions, but not during the test. The marmosets inspected the cup very rarely or not at all during the tests in which either Call A or a background noise was played (when they did so, it was after a delay of at least 160 s). On hearing the playback of Call C, all of the marmosets left the table and inspected (peered into) the cup after a short latency of around 50 s. This is evidence that Call C conveys specific information about the food source and, hence, that it functions as a referential signal, whereas Call A does not. 

Moreover, our results provide some evidence in support of Call C being emitted intentionally, since there was an audience effect; that is, when the marmosets were tested in pairs compared to alone. According to Evans [6], the audience effect is exemplified by fewer calls being produced when the signaller is alone, since the signaller is able to suppress calling in the absence of a receiver. Our results showed the opposite and could be an adaptation of the marmosets’ feeding habits to natural conditions, which demand foraging out-of-sight of conspecifics and a need to signal to group members the discovery of an important food source. Evans’ research [6] was on chicks, which stay in groups and within visual and auditory contact. By contrast, marmosets often forage in dense forests, separately from each other, but remaining within auditory range.

An increase in food calling when out of visual contact with conspecifics has been found in red-bellied tamarins [10]. In contrast, a study of cotton-top tamarins found that the presence or absence of a conspecific did not affect the call rate, although more food calls were given to preferred foods [20]. Di Bitetti [7] found that, in tufted capuchins, it was not the presence or absence of group members that mattered, but the distance from the finder to other conspecifics. Hence, different factors affect food calling in various primate species. In common marmosets, the presence/absence of conspecifics, and the food type were the primary factors that affected food calling.

Producing more food calls when conspecifics are not in close proximity may benefit the social group by sharing food and/or benefit the finder of the food by enhancing vigilance and hence protection from predators [10]. Since common marmosets are prey to a number of species when in their natural environment, the recruitment of group members and social cohesion could benefit survival, as manifested in the group mobbing of predators [30]. Marmosets are also cooperative breeders [31] and the males and females share parenting; hence, sharing information about food with group members would be adaptive.

Although few species have been shown to produce functionally referential food calls [2], our results suggest that at least one food call of common marmosets (Call C) was indeed functionally referential. Other primate species that have been shown to have functionally referential food calls are capuchins [13], rhesus macaques [32,33], chimpanzees [34,35], and Geoffroy’s marmosets [36]. However, as pointed out by Clay et al. [2], since some of the testing procedures used prior training with the food, it is difficult to be sure that, during the playback of the food calls, the test subjects, knowing that food ‘could’ be present, were not responding to the caller’s level of arousal rather than to specific information about the presence of the food. This could have been the case in our study, but we obtained a different result for Call A than for Call C. By presenting banana and waiting until the test animal was actively engaged in feeding on it before the test call was presented as a playback, our marmosets were not simply responding to the level of hunger. In the very least, the feeding marmoset interpreted Call C as signaling that a more preferred food was available. Not entirely distinct from this, but with greater specificity of the call, Call C might have signaled that ‘insects’ were available. Furthermore, the marmoset that was responding to the playback of the C calls did not approach the speaker but went to the site that was associated with mealworms in a different direction and at a different height in the room. In other words, the C calls were interpreted as a signal about the presence of a food type (insects) in a previously learnt location.

It was not possible to say whether Call C was produced solely because the presentation was an insect (mealworm or cricket), or because these were the most preferred foods. However, during the first test of food preference (Experiment 1), the preference for banana was higher than for blueberries, but Call C was never elicited when banana was presented. This supports the conclusion that Call C is given only when insects are seen. 

It is interesting to consider whether the food calls of marmosets are produced spontaneously and ‘honestly’. It has been mentioned above that the stronger the preference for mealworms, the fewer A calls were made while the marmoset had access to the food (lid-off condition). A similar result was found for Call C: the more strongly the marmoset preferred mealworms, the fewer C calls it produced in both the lid-on and lid-off conditions. Hence, while these calls may signal to conspecifics that food is available, some degree of suppression of call production occurs when the food is highly preferred. Deceptive calling about food has been noted in other species; some species that produce food calls use them deceptively to either attract conspecifics when no food is present [37], and others increase the latency to commence food calling when they have found a highly preferred food source, thus allowing the finder to obtain more food without competition from conspecifics [7]. These potential aspects of food calling by common marmosets deserve further research.

## 5. Conclusions

Our study has confirmed that the call that was described previously by Vitale et al. [3], Call A, is a food call. It signals the availability of food, regardless of the food type (fruit or insects). Although Call A is given more frequently when the marmoset is eating alone than when it is paired with a cage-mate, its production can be suppressed, perhaps intentionally, when the food is highly preferred. 

One of the new calls that we investigated, Call B, is emitted only in the presence of food but far less frequently than the other calls, and at lower intensity. Our results suggest that it may be elicited more often by crickets than mealworms or fruit, but further research will be required to determine whether this call is specific for the food-type and whether it is produced intentionally and referentially.

Our main conclusion is the discovery of a new call (Call C) that has specificity for signaling the presence of insects (not fruit) and is a referential signal. This call is produced more frequently when insects can be seen than when they are actually consumed, and when the marmoset is tested alone rather than with its cage-mate. Our results show that C calls are not simply emitted when a preferred food is seen, as there was a negative correlation between the number of C calls that were produced and the strength of preference for mealworms. Also, even in a test in which the marmosets did express a preference for banana, no C calls were emitted. In fact, no C calls were produced in any of the tests in which banana or blueberries were presented.

Our findings provide evidence of the referential function of a food call in common marmosets. They also have implications for improving the captive care of the species.
